# Treatment-Related Adverse Events of Combination Immune Checkpoint Inhibitors: Systematic Review and Meta-Analysis

**DOI:** 10.3389/fonc.2020.00258

**Published:** 2020-03-17

**Authors:** Robin Park, Laercio Lopes, Cagney R. Cristancho, Ivy M. Riano, Anwaar Saeed

**Affiliations:** ^1^MetroWest Medical Center, Tufts University School of Medicine, Framingham, MA, United States; ^2^Division of Medical Oncology, Department of Medicine, Kansas University Cancer Center, Kansas, MO, United States

**Keywords:** combination therapy, immune checkpoint inhibitor, anti-PD-1, anti-PD-L1, anti-CTLA-4, treatment-related adverse events

## Abstract

**Background:** Although clinical practice guidelines for the management of Immune Checkpoint Inhibitor (ICI)-related adverse events have recently been published, precise and nuanced toxicity data for combination ICI therapy are lacking. Therefore, herein we have conducted a systematic review and meta-analysis of published clinical trials on combination ICI to synthesize the treatment-related adverse event (TRAE) profile of combination ICI therapy.

**Methods:** PUBMED, EMBASE, and the Cochrane Database/EBM were searched for eligible studies. Clinical trials evaluating combination immune checkpoint inhibitor therapy in advanced unresectable cancer were included in the analysis based on prespecified criteria. Risk of bias across studies was evaluated using Begg's funnel plot and Egger's regression test. The summary outcomes were pooled risk ratios (RR) and the logit-transformed proportion for incidence data.

**Results:** A total of 18 studies comprising 2,767 patients across 10 cancer types were included in the final analysis. Combination ICI was associated with a slightly higher risk of all-grade adverse events (RR 1.07 [95% CI 1.03–1.11]) and markedly greater risk of grade 3 or higher adverse events (RR 2.21 [95% CI 1.57–3.10]) compared to monotherapy ICI. Subgroup analyses showed significant differences in risk of grade 3 or higher adverse events between treatment types (PD-1 + CTLA-4 and PD-L1 + CTLA-4), among cancer types, and among dosing regimens (N1I3, N3I1, and D20T1). The incidence of all-grade adverse events was 0.905 [95% CI 0.842–0.945], and the ratio of grade 3 or higher events to all-grade adverse events was 0.396 [95% CI 0.315–0.483]. The most common all-grade TRAEs were diarrhea/colitis, fatigue/asthenia, nausea/vomiting, rash, and pruritis.

**Conclusion:** Combination ICI therapy has a significantly different treatment-related adverse event profile compared to monotherapy.

## Introduction

Within a short span of time, with increasingly frequent use of immune checkpoint inhibitors across different types of cancer, knowledge and experience with immune checkpoint inhibitor (ICI)-related adverse events has also been increasingly accumulating (1–6). Based on these accumulated data, clinical practice guidelines have been published to improve the management of these adverse events (7). Several questions remain unanswered, however, on the treatment-related adverse events (TRAEs) of ICI, especially in the setting of combination therapy. For instance, although there is a general consensus that combination ICI therapy results in higher risk of TRAEs compared to ICI monotherapy, data are unclear on whether this risk differs with different ICI combinations or across different cancer types and whether there are notable associations with certain ICI combinations and specific TRAEs (7, 8). Furthermore, it is unclear whether the severity and frequency of adverse events are synergistic or just additive. Therefore, a systematic review of such adverse event data is necessary to guide informed decisions, both in clinical trials and in the clinic, for both clinicians and patients. Herein we conduct a systematic review and meta-analysis of the incidence of all-grade, grade 3 or higher, and individual TRAEs in combination ICI therapy vs. ICI monotherapy with the goal of synthesizing an accurate, precise, and comprehensive toxicity profile.

## Materials and Methods

### Search Strategy

The following meta-analysis is not registered. The search was conducted using PUBMED, EMBASE, and the Cochrane Database/EBM using the following keywords: “PD-1”; “PD-L1”; “CTLA-4”; “pembrolizumab”; “nivolumab”; “tremelimumab”; “ipilimumab”; “atezolizumab”; “durvalumab”; “avelumab.” Only studies published in English from conception to September 28, 2019, were included. Further efforts to identify additional studies included hand-searching of journals and reference lists as well as attempts to contact authors.

### Study Selection

Eligibility assessment was performed independently in a non-blinded standardized manner by two reviewers. Disagreement between the two reviewers was resolved by discussion and consensus. Study inclusion criteria comprised the following: (1) randomized clinical trials; (2) studies in humans; (3) contains adverse event data. Exclusion criteria comprised the following: (1) review articles, meta-analyses, case reports, and case series; (2) conference abstracts and presentations. A data extraction form was developed a priori, and two reviewers in tandem conducted data extraction, with the final results reviewed by a third reviewer. If overlapping data were identified, the most recent or comprehensive study was included. Disagreement was resolved by discussion among the three reviewers. The following information was extracted from each study: (1) study name/clinical trial ID; (2) author; (3) year of publication; (4) cancer type; (5) drugs studied; (6) treatment arms; (7) trial phase; (8) treatment regimen; (6) Common Terminology Criteria for Adverse Events (CTCAE) version used; (9) country where the study was held; (10) adverse event data including total patients, number of total and severe adverse events, and total and severe adverse events for six selected specific TRAEs.

### Statistical Analysis

Pooled risk ratios (RR) and 95% confidence intervals (CI) were calculated using a meta-analytical method that weighed the logarithm of the RR by the function of its variance for each study. Pooled incidence was calculated using the logit-transformed proportion equal to the log of xi/(ni-xi). A random-effects model or a fixed-effects model was chosen based on whether heterogeneity was significant. The Hartung-Knapp adjustment was used to fit the random-effects model, a continuity correction of 0.5 was used in studies with zero cell frequencies, and the Sidik-Jonkman estimator was used to derive tau^2^. Among the selected studies, only those studies containing both combination ICI and monotherapy ICI arms were included for the calculation of the pooled RR, whereas all studies were included in the calculation of the pooled incidence of selected TRAEs. If a study contained more than one monotherapy arm, the combination arm was compared twice separately with each monotherapy arm. Subgroup analysis was conducted based on treatment combination, cancer type, control arm drug, and drug regimen. Publication bias was assessed using Begg's funnel plot and Egger's test, where a *p*-value <0.05 was considered statistically significant. Meta-analysis was performed using the package “metafor” of the R-project.

## Results

### Study Selection

**Figure 1:**
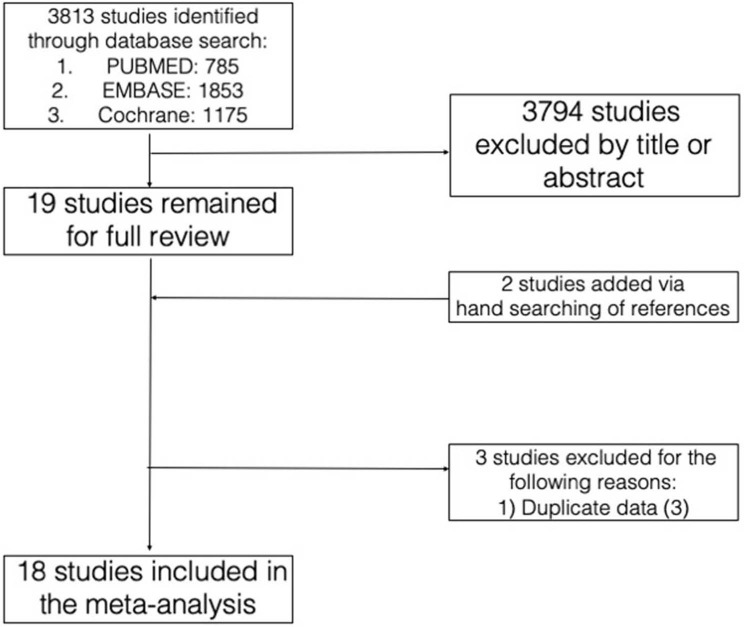
Database search and study selection.

The initial database search yielded 3,813 studies. After selection of studies based on title and abstract review, 19 studies remained for full review, to which two studies were added on the basis of reference search and three studies were excluded based on our pre-specified criteria. The rationale for the addition and exclusion of each study has been summarized (Figure 1).

### Study Characteristics

The final meta-analysis included 18 studies comprised of 2,767 patients (14 anti-PD-1 + anti-CTLA-4; 4 anti-PD-L1 + anti-CTLA-4) across 10 different types of cancer. Among these studies, nine had monotherapy ICI arms (one trial with an anti-PD-L1 arm, one trial with an anti-CTLA-4 arm, one trial with both an anti-PD-1 and an anti-CTLA-4 arm, one trial with both an anti-PD-L1 and an anti-CTLA-4 arm, and the rest with anti-PD-1 arms only). All trials evaluating anti-PD-1 + anti-CTLA-4 were comprised of nivolumab + ipilimumab in the N3I3, N1I3, or N3I1 regimens (nivolumab 3 mg per kilogram + ipilimumab 3 mg per kilogram; nivolumab 1 mg per kilogram + ipilimumab 3 mg per kilogram; nivolumab 3 mg per kilogram + ipilimumab 1 mg per kilogram, respectively) whereas those evaluating anti-PD-L1 + anti-CTLA-4 were comprised of durvalumab + tremelimumab in various dosing regimens. All studies used CTCAE version 4.0 except four that used version 4.03 and one trial that used version 5.0 (Table 1).

**Table 1:** Study characteristics.

**#**	**Study**	**References**	**Cancer**	**Treatment arm**	**Monotherapy arm**	**Phase**	**Regimen**	**Reported Traes (%)**	**CTCAE**	**Number of cases**
1	NCT02374242	Long et al. (11)	Melanoma	PD-1 + CTLA-4	PD-1	II	N1I3^a^	Unspecified	4	35
2	NCT02500797/Alliance A091401	D'Angelo et al. (12)	Sarcoma	PD-1 + CTLA-4	PD-1	II	N1I3^a^	10	4	42
3	NCT02231749/CheckMate 214	Motzer et al. (13)	RCC	PD-1 + CTLA-4	NONE	III	N3I1^a^	15	4	547
4	NCT02477826/CheckMate 227	Hellmann et al. (14)	NSCLC	PD-1 + CTLA-4	PD-1	III	N3I1^a^	15	4	576
5	NCT02320058/CheckMate 204	Tawbi et al. (15)	Melanoma	PD-1 + CTLA-4	NONE	II	N1I3^a^	5	4	94
6	CheckMate 016	Hammers et al. (16)	RCC	PD-1 + CTLA-4	NONE	I	N3I1^a^	Unspecified	4	47
6	CheckMate 016	Hammers et al. (16)	RCC	PD-1 + CTLA-4	NONE	I	N1I3^a^	Unspecified	4	47
6	CheckMate 016	Hammers et al. (16)	RCC	PD-1 + CTLA-4	NONE	I	N3I3^a^	Unspecified	4	6
7	NCT01927419/CheckMate 069	Hodi et al. (17)	Melanoma	PD-1 + CTLA-4	CTLA-4	II	N1I3^a^	Unspecified	4	94
8	NCT01844505/CheckMate 067	Wolchuk et al. (18)	Melanoma	PD-1 + CTLA-4	CTLA-4, PD-1	III	N1I3^a^	5	4	313
9	CheckMate 142	Overman et al. (19)	CRC	PD-1 + CTLA-4	NONE	II	N3I1^a^	10	4	119
10	CheckMate 032	Janjigian et al. (20)	GEC	PD-1 + CTLA-4	PD-1	I/II	N1I3^a^	15	4	49
10	CheckMate 032	Janjigian et al. (20)	GEC	PD-1 + CTLA-4	PD-1	I/II	N3I1^a^	15	4	52
11	NCT02017717/CheckMate 143	Omuro et al. (21)	Glioblastoma	PD-1 + CTLA-4	PD-1	I	N1I3^a^	Unspecified	4	10
11	NCT02017717/CheckMate 143	Omuro et al. (21)	Glioblastoma	PD-1 + CTLA-4	PD-1	I	N3I1^a^	Unspecified	4	20
12	NCT03081923/APACHE	Necchi et al. (22)	Germ cell tumor	PD-L1 + CTLA-4	PD-L1	II	D1500 mg T75 mg^b^	Unspecified	5	11
13	NCT02000947	Antonia et al. (23)	NSCLC	PD-L1 + CTLA-4	NONE	I	D10-20 + T1^b^	10	4.03	56
13	NCT02000947	Antonia et al. (23)	NSCLC	PD-L1 + CTLA-4	NONE	I	D10-20 + T3^b^	10	4.03	34
13	NCT02000947	Antonia et al. (23)	NSCLC	PD-L1 + CTLA-4	NONE	I	D15 + T10^b^	10	4.03	9
14	NCT02319044/CONDOR	Siu et al. (24)	HNSCC	PD-L1 + CTLA-4	CTLA-4, PD-L1	II	D20T1^b^	Unspecified	4.03	133
15	NCT02588131/NIBIT-MESO-1	Calabro et al. (25)	Mesothelioma	PD-L1 + CTLA-4	NONE	II	D20T1^b^	Unspecified	4	40
16	NCT02716272/IFCT-1501 MAPS2	Scherpereel et al. (26)	Mesothelioma	PD-1 + CTLA-4	PD-1	II	N3I1^a^	8	4	61
17	NCT02659059/CheckMate 568	Ready et al. (27)	NSCLC	PD-1 + CTLA-4	NONE	II	N3I1^a^	Unspecified	4	288
18	NCT01454102/CheckMate 012	Hellmann et al. (28)	NSCLC	PD-1 + CTLA-4	NONE	I	N3I1^a^	10	4	38
18	NCT01454102/CheckMate 012	Hellmann et al. (28)	NSCLC	PD-1 + CTLA-4	NONE	I	N3I1^a^	10	4	39

a *N1I3: nivolumab 1 mg/km/kg + ipilimumab 3 mg/kg; N3I1: nivolumab 3 mg/kg + ipilimumab 1 mg/kg; N3I3: nivolumab 3 mg/kg + ipilimumab 3 mg/kg*.

b *D20T1: durvalumab 20 mg/kg + tremelimumab 1 mg/kg; D15T10: durvalumab 10 mg/kg + tremelimumab 10 mg/kg; durvalumab 1,500 mg + tremelimumab 75 mg: durvalumab 1,500 mg + tremelimumab 75 mg*.

### Risk of TRAEs in Combination ICI Compared to Monotherapy ICI

**Figure 2:**
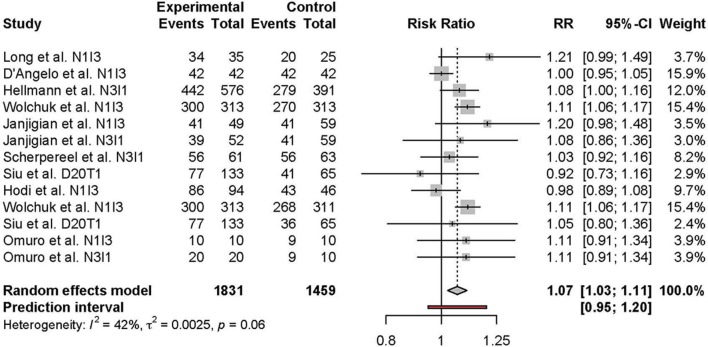
Risk of all-grade adverse events in combination therapy vs. monotherapy.

**Figure 3:**
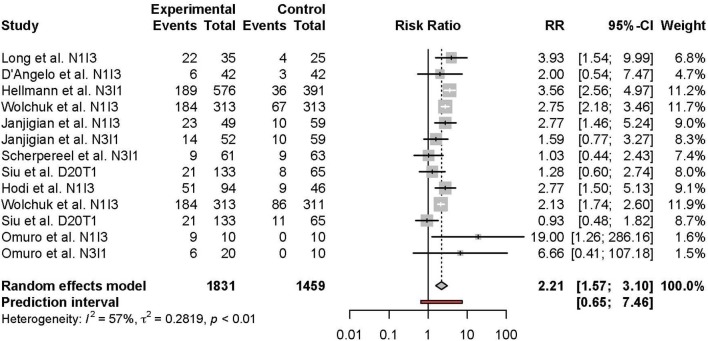
Risk of grade 3 or higher adverse events in combination therapy vs. monotherapy.

Meta-analysis of the comparison between combination and monotherapy ICI included 11 studies comprised of 12 different combination treatment arms (11 anti-PD-1 + anti-CTLA-4 and one anti-PD-L1 + anti-CTLA-4) including seven N1I3, four N3I1, and two D20T1 regimens. Three combination treatment arms were compared to anti-CTLA-4 monotherapy, nine compared to anti-PD-1, and one to anti-PD-L1. The risk of all-grade TRAEs was slightly higher in combination vs. monotherapy ICI (RR 1.07 [95% CI 1.03–1.11]) (Figure 2). In comparison, the risk of grade 3 or higher TRAEs was markedly higher (RR 2.21 [95% CI 1.57–3.10]) (Figure 3).

### Subgroup Analyses

**Figure 4:**
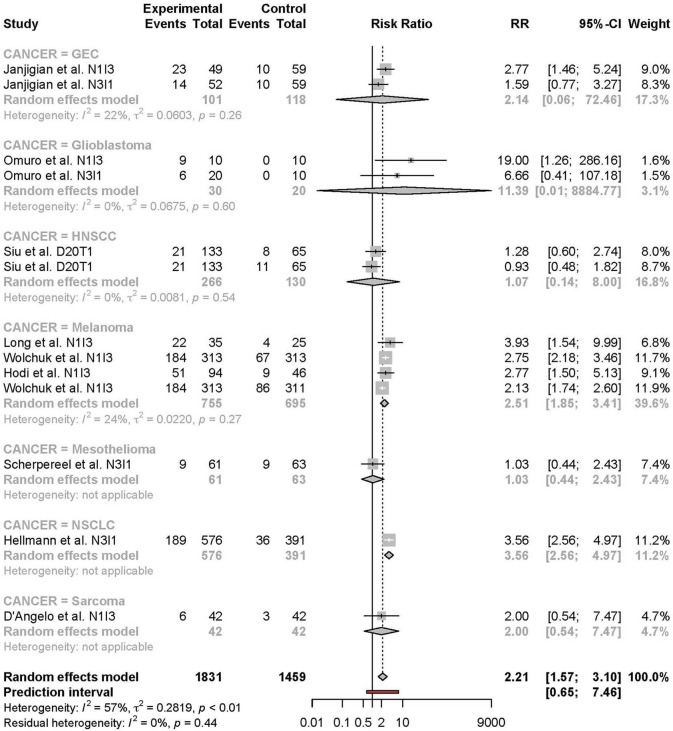
Risk of grade 3 or higher adverse events in combination therapy vs. monotherapy by cancer type.

**Figure 5:**
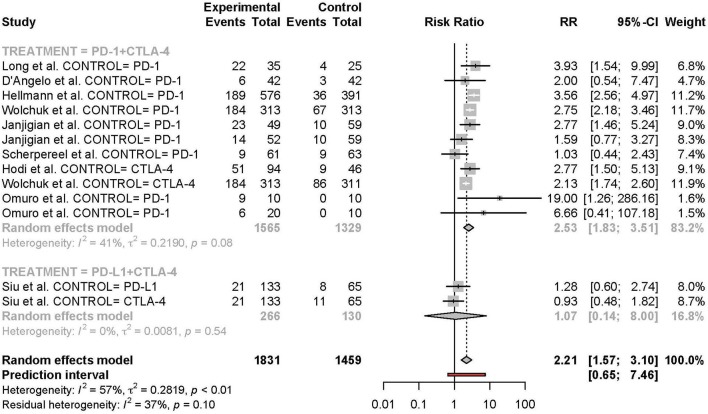
Risk of grade 3 or higher adverse events in combination therapy vs. monotherapy by treatment arm.

**Figure 6:**
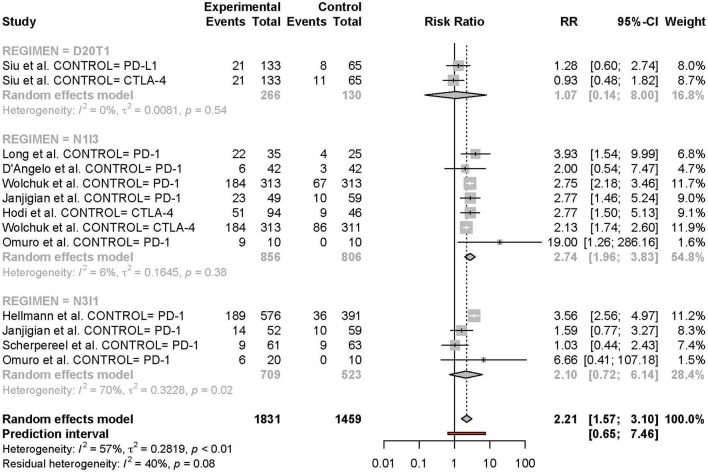
Risk of grade 3 or higher adverse events in combination therapy vs. monotherapy by treatment regimen.

**Figure 7:**
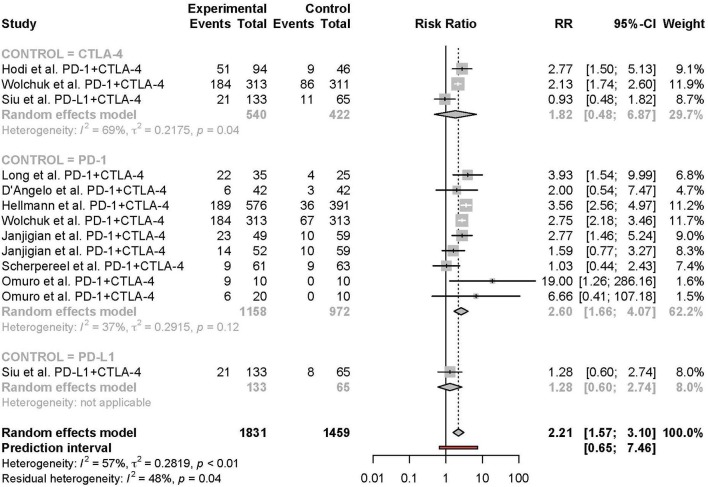
Risk of grade 3 or higher adverse events in combination therapy vs. monotherapy by control arm.

Subgroup analyses were conducted based on cancer type, treatment combination, treatment regimen, and monotherapy arm for grade 3 or 4 adverse events (Figures 4–7). Significant differences were found for all subgroup analyses except analysis by monotherapy arm for grade 3 or 4 adverse events; on the other hand, no significant differences were seen for all-grade adverse events (Table 2).

**Table 2:** Test for subgroup differences.

**Subgroup**	**All-grade adverse events**			**Grade 3 or 4 adverse events**		
	***Q*-value**	**Degree of freedom**	***p*-value**	***Q*-value**	**Degree of freedom**	***p*-value**
Cancer type	10.62	7	0.1560	43.42	6	<0.0001
Treatment combination	0.00	1	0.9799	16.01	1	<0.0001
Monotherapy drug	0.18	3	0.9806	3.03	2	0.2197
Drug regimen	3.49	3	0.3221	20.15	2	<0.0001

### Pooled Incidence of TRAEs

Incidence of all-grade adverse events was 0.905 [95% CI 0.842–0.945], and grade 3 or higher events/all-grade adverse events was 0.396 [95% CI 0.315–0.483]. The most common all-grade TRAEs were diarrhea/colitis, fatigue/asthenia, nausea/vomiting, rash, and pruritis. The most common grade 3 or higher TRAEs in combination ICI were diarrhea/colitis, transaminitis, fatigue/asthenia, lipase elevation/pancreatitis, and nausea/vomiting (Tables 3, 4).

**Table 3:** Pooled incidence of all-grade and grade 3 or higher TRAEs for combination ICI + ICI TRAEs.

**Outcome**	**Incidence (95% CI)**	***I*∧2 (%)**	**Test for heterogeneity**
All grade adverse events	0.905 [0.842–0.945]	95.65	<0.001
Grade 3 or higher adverse events	0.396 [0.315–0.483]	94.74	<0.001
All grade adverse events, PD-1 + CTLA-4	0.911 [0.853–0.948]	93.98	<0.001
Grade 3 or higher adverse events, PD-1 + CTLA-4	0.428 [0.349–0.510]	93.19	<0.001
All grade adverse events, PD-L1 + CTLA-4	0.925 [0.622–0.989]	93.57	<0.001
Grade 3 or higher adverse events, PD-L1 + CTLA-4	0.287 [0.128–0.527]	93.06	<0.001
Grade 3 or higher adverse events, N3I1	0.323 [0.272–0.379]	75.43	<0.001
Grade 3 or higher adverse events, N1I3	0.544 [0.431–0.652]	89.69	<0.001

**Table 4:** Pooled incidence of specific TRAEs.

	**Combination ICI** **+** **ICI**			**PD-1** **+** **CTLA-4**			**PD-L1** **+** **CTLA-4**		
**Outcome**	**Incidence (95% CI)**	***I*∧2 (%)**	**Test for** **heterogeneity**	**Incidence (95% CI)**	***I*∧2 (%)**	**Test for** **heterogeneity**	**Incidence (95% CI)**	***I*∧2 (%)**	**Test for** **heterogeneity**
Cytopenias	0.006 [0.001–0.029]	94.95	<0.001	0.013 [0.003–0.050]	95.07	<0.001	0.009 [0.001–0.083]	80.96	<0.001
Cytopenias, Grade 3 or higher	0.003 [0.001–0.013]	84.16	<0.001	0.004 [0.001–0.018]	85.35	<0.001	0.002 [0.000–0.017]	0	0.673
Diarrhea/Colitis	0.307 [0.219–0.413]	96.46	<0.001	0.334 [0.245–0.436]	95.49	<0.001	0.000 [0.000–1.000]	96.88	0.015
Diarrhea/colitis, Grade 3 or higher	0.067 [0.042–0.106]	89.23	<0.001	0.069 [0.041–0.112]	89.4	<0.001	0.005 [0.001–0.019]	0	0.938
Fatigue/asthenia	0.249 [0.137–0.410]	98.21	<0.001	0.406 [0.306–0.514]	94.96	<0.001	0.000 [0.000–1.000]	96.88	0.015
Fatigue/asthenia, Grade 3 or higher	0.025 [0.017–0.036]	36.9	<0.007	0.029 [0.021–0.040]	31.07	0.019	0.083 [0.041–0.162]	71.42	0.001
Hypophysitis	0.001 [0.000–0.012]	96.75	<0.001	0.002 [0.000–0.021]	97.08	<0.001	0.079 [0.057–0.109]	0	0.28
Hypophysitis, Grade 3 or higher	0.002 [0.000–0.010]	77.53	0.002	0.003 [0.001–0.031]	76.88	<0.001	0.044 [0.021–0.093]	55.79	0.015
Hypothyroidism	0.051 [0.022–0.112]	97.16	<0.001	0.041 [0.012–0.131]	98.48	<0.001	0.070 [0.025–0.177]	82.88	<0.001
Hypothyroidism, Grade 3 or higher	0.002 [0.001–0.007]	53.9	0.319	0.002 [0.001–0.008]	57.46	0.17	0.002 [0.000–0.017]	0	0.673
Lipase elevation/pancreatitis	0.045 [0.019–0.101]	97.13	<0.001	0.067 [0.029–0.145]	97.15	<0.001	0.010 [0.004–0.025]	0	0.731
Lipase elevation/pancreatitis, Grade 3 or higher	0.020 [0.007–0.057]	96.53	<0.001	0.027 [0.009–0.081]	97.15	<0.001	0.083 [0.041–0.162]	71.42	0.001
Nausea/vomiting	0.218 [0.143–0.318]	96.75	<0.001	0.256 [0.172–0.363]	96.34	<0.001	0.116 [0.030–0.356]	93.98	<0.001
Nausea/vomiting, Grade 3 or higher	0.013 [0.008–0.023]	57.49	<0.001	0.018 [0.011–0.028]	49.65	0.004	0.000 [0.000–.0356]	84.24	<0.001
Nephritis/elevated creatinine	0.004 [0.001–0.020]	91.38	<0.001	0.002 [0.000–0.023]	93.96	<0.001	0.002 [0.000–0.017]	0	0.673
Nephritis/elevated creatinine, Grade 3 or higher	0.001 [0.000–0.008]	72.22	0.126	0.001 [0.000–0.009]	73.2	0.031	0.159 [0.055–0.379]	93.33	<0.001
Pneumonitis	0.001 [0.000–0.014]	94.68	<0.001	0.001 [0.000–0.019]	95.11	<0.001	0.002 [0.000–0.017]	0	0.673
Pneumonitis, Grade 3 or higher	0.001 [0.000–0.010]	81.31	<0.004	0.002 [0.000–0.011]	71.22	0.024	0.002 [0.000–0.017]	0	0.673
Pruritis	0.152 [0.099–0.227]	94.82	<0.001	0.182 [0.121–0.265]	94.54	<0.001	0.007 [0.001–0.047]	63.87	0.035
Pruritis, Grade 3 or higher	0.006 [0.003–0.011]	14.02	0.715	0.007 [0.004–0.012]	9.57	0.592	0.004 [0.000–0.107]	84.95	0.001
Rash	0.184 [0.127–0.258]	93.81	<0.001	0.222 [0.155–0.308]	93.81	<0.001	0.007 [0.001–0.047]	63.87	0.035
Rash, Grade 3 or higher	0.008 [0.003–0.021]	72.48	<0.002	0.012 [0.005–0.026]	67.96	0.002	0.009 [0.001–0.119]	89.07	<0.001
Transaminitis	0.098 [0.031–0.269]	98.56	<0.001	0.158 [0.046–0.419]	98.79	<0.001	0.010 [0.004–0.025]	0	0.731
Transaminitis, Grade 3 or higher	0.028 [0.009–0.082]	96.53	<0.001	0.040 [0.013–0.123]	97.11	<0.001	0.012 [0.001–0.152]	81.64	0.011

### Assessment of Bias Across Studies

No significant publication bias was detected per the Rank Correlation Test and Egger's Regression Test for Funnel Plot Asymmetry (Kendall's tau = 0.1003, *p*-value = 0.4652; Reg test: *z*-value 0.9798, *p*-value 0.3272) (Figure S1).

## Discussion

To the best of our knowledge, our meta-analysis is the largest, most comprehensive study on the TRAEs of combination ICI therapy to date. Previous meta-analyses have analyzed the adverse events of combination therapy as a minor subsection of the analysis, for a single tumor type, or for a specific adverse event site (3, 5, 6). Herein, we demonstrate that combination ICI therapy is associated with markedly greater risk of grade 3 or higher adverse events and marginally greater risk of all-grade adverse events compared to monotherapy. Furthermore, our study shows there are significant differences in risk of grade 3 or higher TRAEs when analyzed by type of cancer, treatment combination, and treatment dosing regimen.

For grade 3 or 4 adverse events, subgroup analysis by type of cancer was limited by the small number of studies per type of cancer. Also, the studies included for each type of cancer differed in treatment combination. The risk of grade 3 or higher TRAEs tended to be higher in the melanoma subgroup compared to other cancer type subgroups despite that this subgroup comprised more studies with the N1I3 rather than the N3I1 regimen. This finding is at odds with the notion that anti-CTLA-4 agents in general are known to be more toxic than anti-PD-1 agents (9). This finding may be explained by a feature intrinsic to the tumor type or by between-study differences. Further accumulation of nivolumab + ipilimumab toxicity data in NSCLC is needed to clarify the reason for this difference.

Recent studies have suggested that the clinical response and toxicity of immunotherapy may be altered depending on patient-specific characteristics such as sex and ethnicity (10, 29). Although our analysis did not conduct a subgroup analysis based on patient characteristics due to the limited number of studies, future studies are warranted on the analysis of differences in toxicity in combination ICI therapy based on patient characteristics.

Further subgroup analysis by treatment combination showed a significant difference between the risk of grade 3 or higher TRAE of anti-PD-1 + anti-CTLA-4 vs. anti-PD-L1 + anti-CTLA-4, with higher toxicity in the anti-PD-1-containing group, which is consistent with prior meta-analysis studies of checkpoint inhibitor toxicity. This finding is explained mechanistically by PD-L2 sparing in the context of PD-L1 inhibition. Whether this mechanism translates into lower anti-tumor activity in head-to-head comparisons of anti-PD-1 vs. anti-PD-L1 remains to be answered, and future studies, including clinical trials or network meta-analyses, exploring this question are warranted.

Our pooled incidence analysis suggests that all-grade TRAEs occur in about nine out of 10 patients treated with combination ICI therapy and that four out of ten patients experience grade 3 or higher events. Previous studies suggested that the risk of grade 3 or higher immune-related adverse events ranges from 15 to 42% in anti-CTLA-4 monotherapy, 5 to 10% in anti-PD-1 therapy, and 1 to 7% in anti-PD-L1 therapy (7). The findings of our study are consistent with these previous data, as the incidence of toxicity seems to be driven by the dose of anti-CTLA-4 in the combination therapy regimen. Future studies are necessary to determine whether the toxicity in combination therapy is synergistic or additive.

The results of this study show that an increased risk of adverse events, especially of those that are grade 3 or 4, should be taken into consideration when assessing the benefits of combination therapy. Immune therapy, even as monotherapy, has shown remarkably durable responses in multiple types of cancers, including melanoma, non-small cell lung cancer (NSCLC), renal cell carcinoma, solid-tumors positive for mismatch-repair-deficiency or high in microsatellite-instability (dMMR/MSI-H), and gastric or esophageal cancer (30). However, subsets of patients within these types of cancer and other types of cancer such as mismatch-repair-proficient or microsatellite-instability-low (pMMR/MSI-L) colorectal cancer have primary resistance to monotherapy ICI (31). Therefore, combination therapy, which aims to target multiple immune checkpoints that resistant tumors must have exploited to evade the immune system as part of immune evasion during the process of immune surveillance, is expected to broaden the group of cancer patients who will derive benefit from this effective treatment modality. Furthermore, combination therapy may further increase the benefit derived from monotherapy ICI in tumors already responsive to ICI. Because clinical trials comparing combination ICI to monotherapy ICI with mature clinical outcome data are few-and-far-between, an accurate risk-benefit analysis for combination therapy at this moment is difficult. The data currently available with regards to this question suggest that, in carefully selected populations of certain cancer types, benefit may outweigh risk. For example, metastatic sarcoma (confirmed response with monotherapy 5% vs. combination therapy 16%), metastatic melanoma [2-year overall survival (OS) 63.8 vs. 53.6%], relapsed mesothelioma (12-week DCR 40 vs. 52%) may be those cases (12, 17, 26). Nevertheless, as longer-term OS and progression-free survival (PFS) data become available for combination ICI trials, a more formal risk-benefit analysis should be undertaken.

Our study has several notable strengths. First, this is the first study to our knowledge to have compared different ICI combination regimens for toxicity with sufficient power and potential generalizability across cancer types. Second, all-grade, high-grade, and individual adverse events were all analyzed in this study for in-depth analysis of combination ICI therapy toxicity. Third, this meta-analysis included only randomized clinical trials of combination therapy. Because all randomized trials of combination ICI therapy were completed relatively recently and obviously came after monotherapy trials, reporting of adverse events had evolved compared to the earlier trials of monotherapy and are thus expected to be more accurate and standardized, meaning as a whole that this meta-analysis of only combination therapy trials is comprised of higher quality data compared to previous meta-analyses containing monotherapy trials.

A number of limitations should be noted in this study. Significant heterogeneity is seen across included studies, as with any other meta-analyses. The sources of heterogeneity include but are not limited to cancer types, trial phases, number of previous treatments, criteria or threshold for reporting adverse events, and therapeutic dosages. Some degree of heterogeneity was tolerated for the sake of inclusivity in this study. Furthermore, extensive subgroup analyses were conducted to enhance the sensitivity of this analysis.

In conclusion, combination ICI is associated with significantly higher incidence of severe adverse events compared to ICI monotherapy, and the risks of certain adverse events are markedly increased compared to others. With the publication of ongoing combination ICI therapy trials and new toxicity data, future studies should control for treatment dosages to confirm cancer-type-specific differences in toxicity and clarify PD-L1-containing regimen toxicity. Furthermore, analysis of therapeutic combinations not studied herein should be carried out, especially combinations containing pembrolizumab, given its growing number of indications in different cancer types.

## Data Availability Statement

All datasets generated for this study are included in the article/Supplementary Material.

## Author Contributions

RP contributed substantially to the conception, design of study, acquisition of data, quality control, data analysis and interpretation, statistical analysis, manuscript preparation, editing, and review. LL contributed substantially to the data acquisition, quality control of data and algorithms. CC and IR contributed substantially to the data acquisition and quality control. AS contributed substantially to the conception and design of study, manuscript preparation, editing, and review.

### Conflict of Interest

AS reports research funding (to institution) from AstraZeneca, Exelixis, Bristol Myers Squibb, Clovis, and Merck and advisory board/consulting fees from Bristol Myers Squibb, AstraZeneca, and Exelixis. The remaining authors declare that the research was conducted in the absence of any commercial or financial relationship that could be construed as a potential conflict of interest.

## Supplementary Material

Figure S1 Begg's funnel plot.
