# Supplementary figures and images for: Treatment-Related Adverse Events of Combination Immune Checkpoint Inhibitors: Systematic Review and Meta-Analysis
Source: Front Oncol. 2020 Mar 17;10:258. doi: 10.3389/fonc.2020.00258 (PMC7090167; doi:10.3389/fonc.2020.00258)

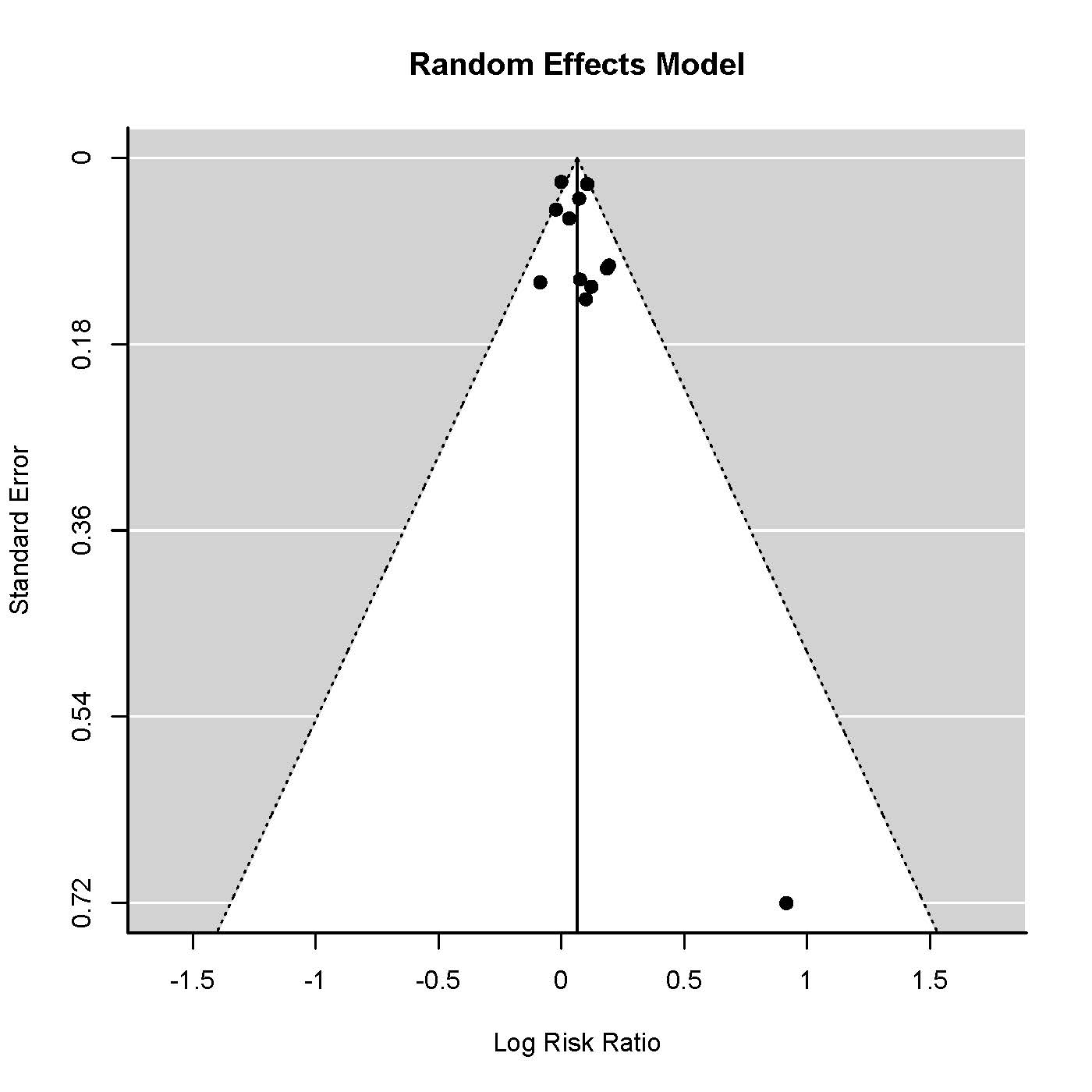

Supplement: Figure S1 — Begg's funnel plot. [file Image_1.jpeg]
